# The efficacy of moxibustion and acupuncture therapy for ankylosing spondylitis: A protocol for an overview of systematic reviews and meta-analysis

**DOI:** 10.1097/MD.0000000000025179

**Published:** 2021-04-16

**Authors:** Qingyuan Zhu, Jun Chen, Jun Xiong, Lunbin Lu, Siyuan Zhu, Zhiying Zhong, Genhua Tang, Xingchen Zhou, Han Guo

**Affiliations:** a Nanchang Hongdu Hospital of Traditional Chinese Medicine; b Jiangxi University of Traditional Chinese Medicine; c The Affiliated Hospital of Jiangxi University of Traditional Chinese Medicine, Nanchang, China.

**Keywords:** acupuncture, ankylosing spondylitis, AS, du-moxibustion, GRADE, moxibustion, overview, protocol

## Abstract

**Background:**

Ankylosing spondylitis is a complex and progressive autoimmune inflammatory disease with a worldwide prevalence ranging up to 0.9%. Several systematic reviews and meta-analyses of traditional Chinese medicine alternative therapies, such as acupuncture or moxibustion, have demonstrated the effectiveness of moxibustion and acupuncture in the treatment of ankylosing spondylitis. However, there is no relevant literature to comprehensively evaluate the evidence. The purpose of this overview is to synthesize and evaluate the reliability of evidence generated in the systematic review (SR) and meta-analysis of moxibustion and acupuncture as a primary or complementary therapy for patients with ankylosing spondylitis.

**Methods:**

PubMed, EMBASE, the Cochrane Central Register of Controlled Trials, Chinese National Knowledge Infrastructure, Chinese VIP Information, Wanfang Database, and Chinese Biomedical Literature Database were searched for systematic reviews and meta-analysis that review the efficacy of acupuncture or moxibustion as the primary treatment for patients with Ankylosing Spondylitis. The literature published before August 2020 will be selected. Additionally, the relevant SRs and meta-analyses that unpublished or ongoing will be searched in PROSPERO and INPLASY. The methodological guidelines for overviews will be used to review and extract data by 2 reviewers, and their will do it independently. Methodology quality will be analyzed by the assessment of multiple systematic reviews-2and the risk of bias by POBIS. For the included studies, we will adopt the following results as primary evaluation indicators: effective rate, visual analogue scale and bath AS disease activity index. Reviewers will assess the certainty of evidence by Grading of Recommendations Assessment, Development and Evaluation.

**Results:**

The results will be published in a peer-reviewed journal.

**Conclusion:**

This overview will provide comprehensive evidence of moxibustion and acupuncture for patients with Ankylosing Spondylitis.

## 1 Introduction

### 1.1 Description of the condition

Ankylosing spondylitis (AS), a common inflammatory rheumatic disease that affects primarily the sacroiliac joints and the axial skeleton,^[1,2]^ is characterized by inflammatory erosive osteopenia and unusual bony overgrowth.^[3]^ The adverse effects of AS not only include physical pain, impaired function, and reduced quality of life, but may also have an impact on employment prospects.^[4]^

A number of epidemiological studies on AS show that the incidence of AS has a great correlation with gender and age.^[1,5–7]^ In different countries, male patients constituted a 2 to 6.1-fold higher number of patients than female, and had a significantly higher mean age at diagnosis.^[5]^ An recent epidemiological survey has shown that the AS prevalence rate in China was 0.29% and continues to increase.^[6]^ The incidence of AS in the United States has been reported from 0.2 to 0.55 in related epidemiological studies.^[3,8,9]^ AS tends to occur in the second and third decades of life,^[2]^ as a result, the quality of life of patients is reduced, and the employment prospects of patients will also have a very negative impact.^[4]^ In a series of economic evaluation studies on AS conducted in the United States, AS represents a substantial burden both on society and individuals with AS. In the total cost of AS, the indirect costs caused by AS account for the majority.^[4,10–13]^ In addition to the financial burden, AS also brings other extra-articular disease burden, such as acute anterior uveitis , cardiac conduction system abnormalities, aortic regurgitation, neurological sequelae, and amyloidosis.^[2,14–16]^ The etiology and pathogenesis of AS remain unclear, but the most convincing hypothesis is immune mediation mechanism, including several cytokines like tumor necrosis factor (TNF), genetic factors, interaction between T cell response, and environmental factors.^[17,18]^ Besides, some relevant genetic studies have confirmed that the incidence of AS has a strong correlation with HLA-B27 that is found in about 90% of patients with AS.^[19–22]^

The basic principles of treat for patients with AS should be based on the presentation of the disease, severity of symptoms, and some other factors. The best treatment needs to combine the nonpharmacological with pharmacological treatment methods. Nonpharmacological therapy consists of spa treatment,^[23]^ physical therapy, acupuncture, as well as moxibustion. Pharmacological therapy is the main treatment for AS, in current studies and guidelines, nonsteroidal anti-inflammatory drugs (NSAIDs) are considered the first choice for patients with AS, which works through inhibiting prostaglandin synthase from relieving pain, stiffness, and inflammation.^[24]^ However, NSAIDs are known to have side effects, such as gastrointestinal adverse reactions and adverse effects on the cardiovascular system, which could restrict their use.^[25,26]^ In addition to the side effects, some patients with AS may be insensitive to NSAIDs, in the event of this, disease-modifying anti-rheumatic drugs are the second-line therapy recommended for patients with AS.^[28]^ Meanwhile, TNF-α inhibitors (anti-TNFs) also play a significant role that suppresses the immune system and reduces the inflammation in the joints for the patients with low sensitivity to NSAIDs.^[25,29]^

Although the majority of patients who suffered from AS experienced different relief of the disease after the use of aforementioned drugs, but those are palliative measures and do not change disease course.^[2]^ At the same time, the financial burden of taking medication forces many patients to seek other effective and accessible treatments.

### 1.2 Description of the intervention

Acupuncture and moxibustion, as traditional Chinese medicine therapies, are also widely accepted complementary and substitution therapies in the world. It has already been confirmed that can effectively improve the symptoms of AS patients, and has been widely used in rheumatic immune system diseases.^[30]^

Acupuncture refers to the treatment of alleviating pain or other symptoms of a disease by inserting a needle into an acupuncture point under the technical guidance of an experienced doctor to produce electrophysiological changes. Acupuncture includes electro-acupuncture, fire-needle, acupoint injection, auricular needle, etc.

Several studies have reported that needle insertion at acupoints not only can improve the blood indices with an increased volume of blood flow but also effectively reduce the release of local inflammatory mediators, and reduce inflammation and pain.^[31,32]^

The mechanism of moxibustion is based on modem theory of thermal effect.

Moxibustion is a characteristic therapy that applies heat to acupoints by burning moxa or herbal material at the acupoints to be stimulated and the heat would be transferred to the skin and recognized by the thermal sensory receptors as invasive stimulation.^[33]^ When the thermal sensory receptors are activated by the heat thermal stimuli, the sensory signals are transmitted to the central nervous system via nerve fibers, thus producing therapeutic effects.^[34]^ The mechanism of moxibustion has been reported by some systematic reviews (SRs) and meta-analysis published in Chinese that moxibustion can effectively regulate the immune imbalance and inflammatory reaction in patients with AS.^[35,36]^

The use of moxibustion, as an alternative therapy, in the treatment of patients with AS, not only can avoid the side effects of oral drugs, but also can reduce the patient's physical and financial burden.

### 1.3 The reason to perform this overview

Recently, plenty of studies have been performed to examine the effect of acupuncture and moxibustion treatments on AS. Some SR and meta-analysis have shown that acupuncture and moxibustion can provide some positive impact for patients with AS mainly involve regulate the immune imbalance and inflammatory reaction to reduce joint pain and systemic symptoms. However, the evidence supporting its effectiveness and safety is still limited.

In this overview, the goal was to review existing or not published but completed SRs and meta-analyses and to gather the information of the evidence for a comprehensive synthesis of evidence, and evaluate the level of evidence with GREAD to provide reliable evidence for clinical workforce and help AS patients seek more reasonable treatments.

## 2 Methods

### 2.1 Registration

This overview will be reported according to the Preferred Reporting Items for Systematic Reviews and Meta-analyses Protocols (PRISMA-P).^[37]^ It is registered in the INPLASY (registration number, INPLASY202080032; https://inplasy.com/inplasy-2020-8-0035/).

### 2.2 Inclusion criteria for this overview

PICOS will be applied, including population, intervention, comparison, outcome, and study.

#### 2.2.1 Types of studies

All relevant SRs and meta-analyses of randomized controlled trials (RCTs) in which acupuncture or moxibustion was utilized as the treatment for the AS. No restrictions are on country but language will be limited on English and Chinese.

#### 2.2.2 Participants

Study participants are the patients with all types of AS and will not be restricted to age, sex, or nationality.

#### 2.2.3 Intervention

AS treatments include acupuncture (it includes electro-acupuncture, fire needle, acupoint injection, transcutaneous electrical acupoint stimulation, etc) and moxibustion (it includes Du-moxibusition/long snake moxibustion, thermal moxibustion, etc). We also included trials that compared acupuncture or moxibustion combined with another active treatment with other active treatments alone.

#### 2.2.4 Comparator

The selected SRs or meta-analyses should testify that the interventions were compared with a control group composed of placebo, sham acupuncture/moxibustion, no treatment, or other active therapies.

#### 2.2.5 Outcomes

Primary outcomes: effective rate, visual analogue scale, and bath AS disease activity index.

Secondary outcomes: finger-to-floor distance, occiput to wall distance, CRP, erythrocyte sedimentation rate, and side effects.

### 2.3 Search strategy

Two investigators will retrieve the relevant SRs and meta-analyses in the following databases: PubMed, Embase, the Cochrane Library, CNKI, Chinese VIP information, Wanfang Database, and CBM, from inception until August 2020. The language will be restricted to Chinese and English. A comprehensive search strategy will be conducted, MeSH items and free words will be searched synchronously. The following search terms will be used: ankylosing spondylitis, spondylitis, ankylosing, ankylosing spondylarthritis, acupuncture, moxibustion, du-moxibustion, long snake moxibustion, systematic review, meta-analysis, etc. The preliminary search strategy for PubMed is presented in Table 1.

**Table 1:** Search strategy (PubMed).

Order	Strategy
#1	Search “Spondylitis, Ankylosing” [Mesh]
#2	Search ((((Ankylosing Spondylitis[Title/Abstract]) OR Ankylosing Spondylarthritis [Title/Abstract]) OR Spondyloarthritis Ankylopoietica [Title/Abstract]) OR Spondylarthritides, Ankylosing[Title/Abstract]
#3	#1 OR #2
#4	Search “Acupuncture” [Mesh] OR “Moxibustion” [Mesh]
#5	Search (((((Acupuncture[Title/Abstract]) OR Acupuncture Therapy[Title/Abstract]) OR Moxibustion[Title/Abstract]) OR Du moxibustion[Title/Abstract]) OR Long snake moxibustion[Title/Abstract])
#6	#4 AND #5
#7	Search “Systematic Reviews as Topic” [Mesh] OR “Systematic Review” [Publication Type]
#8	Search (((((Systematic Reviews[Title/Abstract]) OR systematic review[Title/Abstract]) OR SR[Title/Abstract]) OR SRs[Title/Abstract]) OR review[Title/Abstract]
#9	Search “Meta-Analysis as Topic” [Mesh] OR“Meta-Analysis” [Publication Type]
#10	#7 OR #8 OR #9
#11	#3 AND #6 AND #10

### 2.4 Studies selection

After searching, the duplicated studies will be removed initially from the retrieved studies by Endnote(X9). And then, 2 independent reviewers (SZ and LL) will screen titles, abstracts, and keywords of all retrieved studies for candidates according to the inclusion and exclusion criteria, we will obtain the full text of all possibly relevant studies. Excluded studies will be recorded with explanations. If it is uncertain whether to adopt because of the lack of information, GHT will try to contact authors of the original reports to obtain the information of lost.

**Figure 1:**
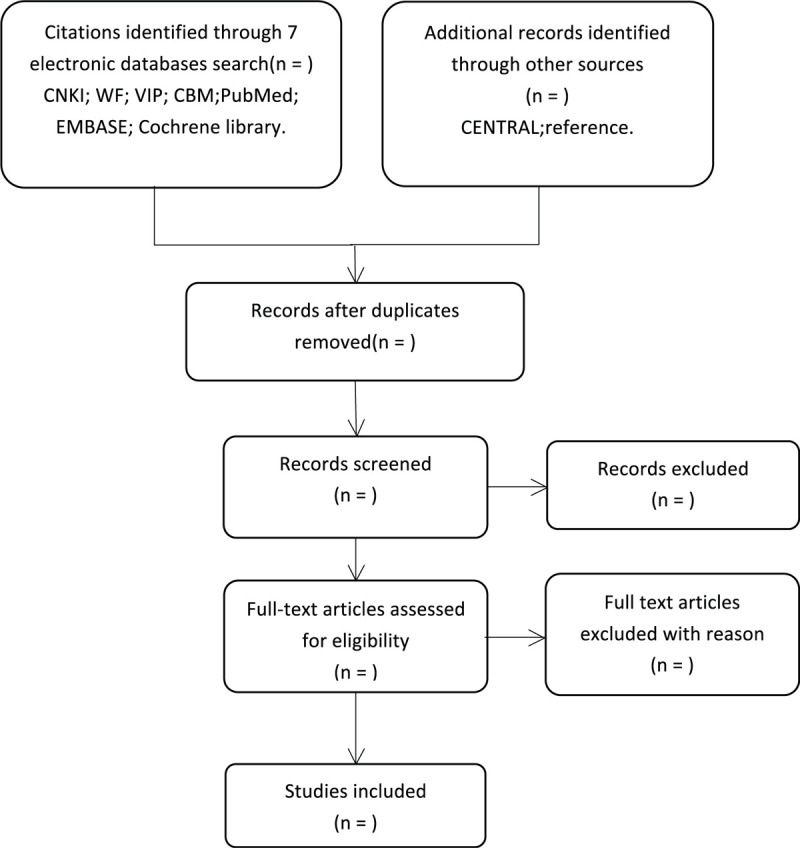
Flowchart of literature selection.

During the procedure, disagreements will be resolved by discussion or consensus with the third reviewer (JX). Study selection will be performed in accordance with the PRISMA flowchart (Fig. 1).

### 2.5 Data extraction

To ensure that necessary information would not be lost, each of the SRs and meta-analyses will be submitted to data extraction by 2 independent reviewers (JC and LL); the following data will be extracted:

1. Study characteristics: author, country, year of publication, study design, and database.
2. Population characteristics: sex, age, baseline diseases, and sample size.
3. Methodological characteristics: information sources, intervention(s), comparison(s), bias assessment, and funding, etc.

If there are any differences, we will resolve them by mutual discussion or by consensus with the third reviewer (JX).

### 2.6 Data analysis

#### 2.6.1 Assessment of risk of bias

Two authors (SZ and LL) evaluated the quality and risk of bias of the included SR/meta-analysis independently using the Risk of Bias in Systematic reviews tool and PRISMA.^[38]^ A consensus is reached by 2 reviewers through discussion, and independent decisions are made by experts (JX) if necessary.

#### 2.6.2 Assessment of methodological and reporting quality

The tool assessment of multiple systematic reviews-2 (AMSTAR-2)^[39]^ will be referenced to assess the methodological quality of the SRs and meta-analyses by 2 separate reviewers (ZZ and GT). If disagreements occur, it would be solved through discussion between 2 reviewers or consulting the expert's (JX) decision.

#### 2.6.3 Assessment of evidence quality (GRADE)

The quality of evidence will be evaluated using the GRADE approach.^[40]^ The evidence quality of all outcomes will be rated on 4 levels (high, medium, low, or very low). Two reviewers (XZ and HG) will conduct the assessment process separately and describe in detail the reasons for downgraded or upgraded outcomes affecting the quality of evidence to guarantee the reliability and transparency of results.

#### 2.6.4 Dealing with missing data

If the specific information is not reported, we will attempt to contact the original author for relevant information. If the required information is not available, it will be explained in the article. Then, the analysis will rely on existing data, and discuss the potential impact of missing information.

### 2.7 Data synthesis

The original studies, possibly, would be overlapped in the studies that we included. For this situation, we will take corresponding measures to solve the overlapping before data synthesis. If the major RCT are overlapping more than two-thirds, the higher quality review will be selected; conversely, both reviews will be retained.

Considering that only carry out qualitative evaluation instead of quantitative analysis, if produce significant overlaps and biased results.

The random-effects model (I^2^≥50%) or fixed-effects model (I^2^ < 50%) will be selected according to the heterogeneity levels of the included SRs and meta-analyses. If the I^2^ value is higher than 70%, the heterogeneity will be explored through discussion with the review team.

### 2.8 Subgroup analysis and investigation of heterogeneity

If large heterogeneity is observed after data synthesis of the included systematic reviews and meta-analyses, subgroup analysis or sensitivity analysis will be performed to find the source of heterogeneity, and subgroups will be conducted according to the different types of AS, intervention measures, or types of outcomes.

### 2.9 Assessment of reporting biases

The publication bias will be assessed through funnel plots and Egger test if sufficient numbers of SRs and meta-analyses are included. All eligible trials will be included, regardless of their methodological quality.

### 2.10 Ethics and dissemination

This study is based on published systematic reviews and meta-analyse, and does not involve personal data. Therefore, the ethical approvals and patient consent are not necessary. The articles in this overview will be submitted to peer-reviewed journals for publication according to the PRISMA-P guideline.

## 3 Discussion

AS is a chronic, debilitating condition that causes severe back pain. It mainly affects the sacroiliac joints and axial skeleton, leading to spinal stiffness. AS affects 0.9% of the world's population, and most of the patients are men under 30 years old. AS is characterized by insidious onset, long disease course, and high disability rate, and has gradually become a serious public health problem.^[41]^

Despite some SRs and meta-analyses about acupuncture and moxibustion for AS, the efficacy and safety of acupuncture and moxibustion treatment for AS has not been reviewed. At present, there is no relevant literature to evaluate the methods and report quality of these studies. This overview will systematically use the AMSTAR-2 and GRADE to assess the quality of available evidence.

This overview, meanwhile, presented to have some limitations, mostly due to the quality of the original research. First, primary studies with partial overlap between included SRs and meta-analyses, and the synthetic result may be affected by duplicate clinical trials. Besides, some necessary information may be missing in the included studies, because the retrieval of literature is limited to Chinese and English databases. But this overview is still expected to provide a better method for the treatment of AS and provide reliable evidence for clinical staff.

## Acknowledgments

The authors thank the following people who either provided feedback on the protocol or supported the development of the methods: Jun Chen^1^, Jun Xiong^2^, Lunbin Lu^3^, Genhua Tang^4^, Siyuan Zhu^5^, Zhiying Zhong^6^, Xingchen Zhou^7^, Han Guo^8^.

Authors’ information: ^1^Department of Acupuncture and Moxibustion, Graduate College, Jiangxi University of Traditional Chinese Medicine, Nanchang, Jiangxi province, China. ^2^Department of acupuncture and Moxibustion, The Affiliated Hospital of Jiangxi University of Traditional Chinese Medicine, Nanchang, China. ^3^Department of Acupuncture and Moxibustion, Graduate College, Jiangxi University of Traditional Chinese Medicine, Nanchang, Jiangxi province, China. ^4^Department of Acupuncture and Moxibustion, Graduate College, Jiangxi University of Traditional Chinese Medicine, Nanchang, Jiangxi province, China. ^5^Department of Acupuncture and Moxibustion, Graduate College, Jiangxi University of Traditional Chinese Medicine, Nanchang, Jiangxi province, China. ^6^Department of Acupuncture and Moxibustion, Graduate College, Jiangxi University of Traditional Chinese Medicine, Nanchang, Jiangxi province, China. ^7^Department of acupuncture and Moxibustion, The Affiliated Hospital of Jiangxi University of Traditional Chinese Medicine, Nanchang, China. ^8^Department of acupuncture and Moxibustion, The Affiliated Hospital of Jiangxi University of Traditional Chinese Medicine, Nanchang, China.

## Author contributions

All authors have read and approved the publication of the protocol.

**Conceptualization:** Qingyuan Zhu, Jun Chen, Jun Xiong

**Data curation:** Jun Chen, Lunbin Lu, Siyuan Zhu, Genhua Tang, Zhiying Zhong, Xingchen Zhou, Han Guo.

**Formal analysis:** Jun Chen, Lunbin Lu.

**Investigation:** Jun Xiong, Jun Chen.

**Methodology:** Jun Chen, Siyuan Zhu, Lunbin Lu.

**Software:** Genhua Tang, Zhiying Zhong.

**Supervision:** Jun Xiong, Xingchen Zhou.

**Writing – original draft:** Qingyuan Zhu, Jun Chen, Jun Xiong, Lunbin Lu, Siyuan Zhu.

**Writing – review & editing:** Jun Xiong, Xingchen Zhou, Lunbin Lu, Siyuan Zhu, Han Guo.
